# Biomimetic double-layered electrospun nanofibrous scaffold with mussel adhesive protein coating and TGF-β3 encapsulation for enhanced tendon-bone healing in rotator cuff tears

**DOI:** 10.1016/j.mtbio.2026.103160

**Published:** 2026-04-27

**Authors:** Sheng Fang, Yiming Wang, Huan Li, Hanwen Li, Zhuang Zhu, Huan Wang, Feng Han, Shenghao Wang, Dachuan Liu, Jiaying Li, Chenxu Zhu, Qifan Yu, Li Dong, Chen Cui, Zhaofan zhang, Jinbo Liu, Bin Li, Song Chen

**Affiliations:** a Department of Articular Orthopedics, The Third Affiliated Hospital of Soochow University, Changzhou, Jiangsu, 213003, China; b Changzhou First People's Hospital, Changzhou Medical Center, Nanjing Medical University, Changzhou, Jiangsu, 213000, China; c Medical 3D Printing Center, Orthopedic Institute, Department of Orthopedic Surgery, The First Affiliated Hospital, School of Basic Medical Sciences, Interdisciplinary Innovation Center for Nanomedicine, MOE Key Laboratory of Geriatric Diseases and Immunology, Suzhou Medical College, Soochow University, Suzhou, Jiangsu, 215000, China; d Division of Spine Surgery, Department of Orthopedic Surgery, Nanjing Drum Tower Hospital, Affiliated Hospital of Medical School, Nanjing University, Nanjing, Jiangsu, 210000, China

**Keywords:** Tendon-bone interface, Rotator cuff tear, Mussel adhesive protein, Electrospun scaffold, Enthesis

## Abstract

Poor tendon-bone healing is a challenging issue and contributes to the high retear rate following rotator cuff tear (RCT) repair. The complex multiple tissue structure and limited chondrogenic capacity at the tendon-bone interface hinder effective regeneration and restoration of the enthesis. In this study, we developed a double-layered biomimetic nanofibrous scaffold encapsulating TGF-β3, further functionalized with a mussel adhesive protein (MAP) coating, to target the reconstruction of the torn rotator cuff's enthesis. The unique double-layer structure features distinct fibrous arrangements in each layer, mimicking the heterogeneous extracellular matrix structures of tendon and bone. This design provided a biomimetic environment conducive to the ingrowth of multiple tissues at the interface. In vitro studies demonstrated that the MAP-coated scaffold exhibited excellent biocompatibility and enhanced cell adhesion, facilitating tendon-bone interfacial integration. Moreover, the sustained release of TGF-β3 promoted stem cell recruitment and chondrogenic differentiation, as demonstrated both in vitro and in vivo. RNA-sequencing revealed that PI3K-Akt signaling pathway might be associated with the regulatory effects of the scaffold. In a rat RCT model, the composite scaffold significantly enhanced cartilage regeneration at the tendon-bone interface, restoring both enthesis structure and biomechanical properties. Therefore, the composite scaffold represents a promising strategy for improving tendon-bone healing and advancing interfacial tissue engineering in rotator cuff repair.

## Highlights

• Complex structure and limited chondrogenesis lead to poor tendon-bone healing.
• The double-layered scaffold offers a biomimetic ECM for multiple tissue growth.
• MAP coating enhances the scaffold's biocompatibility and adhesion properties.
• Sustained release of TGF-β3 recruits stem cells and promotes chondrogenesis.
• The composite scaffold reconstructs the native enthesis structure of rotator cuff.

## Graphical abstract

**Figure:**
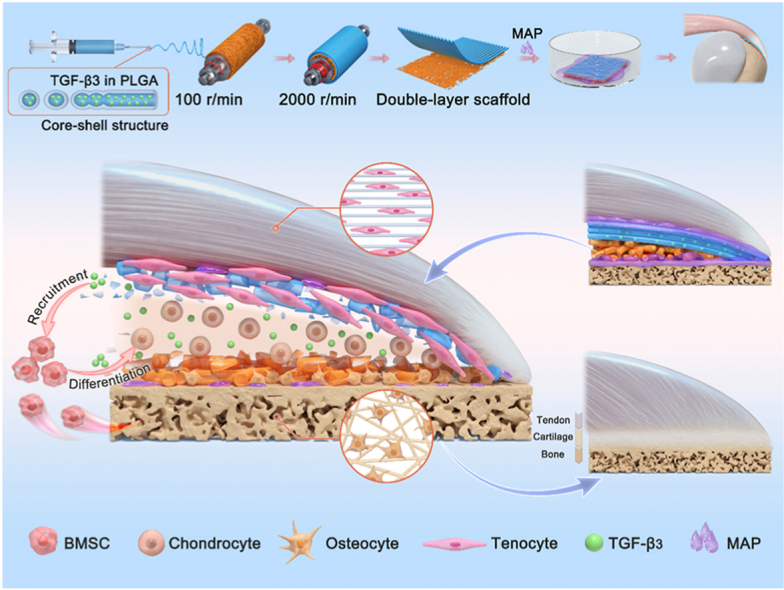

## 1 Introduction

Rotator cuff tear (RCT) is one of the most prevalent shoulder disorders, marked by substantial pain and dysfunction, particularly affecting the elderly and adults [1]. The primary approach to treating RCT is arthroscopic repair, but post-surgery retear rates are reported as 20% to 60% [2,3]. The predominant factor contributing to retears is attributed to poor tendon-bone healing, characterized by insufficient cartilage regeneration followed by scar formation at the tendon-bone interface [2,[4], [5], [6]]. The natural structure of the tendon-bone junction (enthesis) in the rotator cuff comprises tendon, cartilage, and bone, forming a gradient multilayered structure [7,8]. This gradient structure effectively facilitates load transfer and diminishes stress concentrations at the tendon-bone interface [[7], [8], [9]]. In contrast, scar tissue falls significantly short in mechanical strength and stress transfer compared to the native enthesis, thereby increasing the risk of retear at the repaired interface. Hence, the key determinant for successful tendon-bone healing is the restoration of the enthesis structure, with a particular emphasis on the cartilage transition zone [[7], [8], [9], [10], [11]]. Despite its critical significance, the challenge persists in devising effective methods to reconstruct the enthesis structure at the tendon-bone interface.

Given the limited intrinsic healing capacity, tissue engineering has been employed to enhance tendon-bone healing [[12], [13], [14]]. Various biomaterials, such as hydrogels [[15], [16], [17]], electrospun fibrous scaffolds [4,18], and decellularized extracellular matrix (ECM) scaffolds [19,20], have been introduced for repairing RCTs. The ideal scaffold should exhibit following characteristics: nontoxicity, favorable biocompatibility, biodegradability, and biomimicry, acting as a temporary ECM to support cell proliferation and differentiation [4,14,21,22]. Electrospun scaffolds, with their nanoscale fiber structure, high porosity, and large specific surface areas, closely resembling the physical structure of the native ECM, have been widespread use in tissue engineering, including RCT repair [[23], [24], [25], [26]]. However, it is worth to note that the tendon-bone interface, unlike other single-tissue interfaces, involves both soft and hard tissues with markedly dissimilar ECM structures. Some literature have shown that collagen fibers, the main components maintaining the ECM's structure, are aligned and parallel in tendon tissue, whereas they are random in bone tissue at the interface [7,8]. As a result, conventional scaffolds featuring a single structure prove inadequate in simultaneously replicating dual tissue structures. In this study, we engineered a double-layered biomimetic electrospun scaffold, incorporating varied fiber arrangements in distinct layers. The upper layer, with an aligned fiber arrangement, is able to provide a biomimetic ECM structure for the tendon, while the lower layer, with a random fiber arrangement, mimics the ECM structure of bone. We hypothesized that the double-layered scaffold could resolve the mismatch between the scaffold and the tendon-bone interface, establishing a biomimetic environment that promoted the regeneration of multiple tissues.

Poly lactic-co-glycolic acid (PLGA) is an FDA-approved, non-toxic, degradable synthetic co-polymer widely utilized in biomedical applications [27,28]. However, PLGA, being a hydrophobic material, is less conducive to cell and tissue adhesion. Mussel adhesive protein (MAP), secreted from the foot gland of marine mussels, provides mussels with remarkable adherence to various surfaces, even in aqueous environments. MAP demonstrates low toxicity, good biocompatibility and robust adhesion owing to its high content of 3,4-dihydroxyphenylalanine (DOPA) and lysine [29,30]. MAP can enhance cell adhesion, proliferation, tissue integration and regeneration, thus widely used in bioregenerative medicine [[29], [30], [31], [32], [33]]. Literature reported MAP-functionalized electrospun nanofibers and hydrogel accelerate wound repair, while MAP coatings on titanium implants improve osseointegration [[31], [32], [33]]. In our current study, we utilized MAP as a surface coating to modify the hydrophilicity and adhesion properties of the PLGA scaffold. Our hypothesis was that the MAP-coated scaffold could improve the binding between tendon and bone, enhancing cell adhesion and proliferation. This, in turn, would promote early-stage tendon-bone conjunction and regeneration at the interface.

Revitalizing the cartilage transition zone stands as the most pivotal and formidable obstacle in reinstating the enthesis of the rotator cuff. This challenge emanates from the scarcity of chondrocytes and limited formation of cartilage matrix within the tendon-bone region [13,14,34]. Several cytokines or drugs with pro-chondrogenic effects have been reported to enhance tendon-bone healing [5,10,34]. Transforming growth factor-β3 (TGF-β3), acknowledged for its prominent pro-chondrogenic ability [[35], [36], [37]], emerges as the most promising candidate for regenerating the cartilage transition zone to rebuilt the enthesis structure. However, TGF-β3 is a hydrophilic protein with a short active half-life, making it susceptible to denaturation. Consequently, a reliable and sustained delivery system is essential to maintain the bioactivity and effective concentrations of TGF-β3 at the interface. Literature reviewed the extensive use of electrospun scaffolds for growth factors delivery and a core-shell structure scaffold can achieve sustained or controlled release of growth factors while ensuring their biological activity [[38], [39], [40], [41]]. Therefore, we employed the emulsion electrospinning technique to encapsulate TGF-β3 into PLGA nanofibers, creating a core-shell structure within the scaffold fibers. We hypothesized that this core-shell structure would preserve the bioactivity and ensure the sustained release of TGF-β3, thereby inducing cartilage regeneration at the tendon-bone interface.

**Scheme 1:**
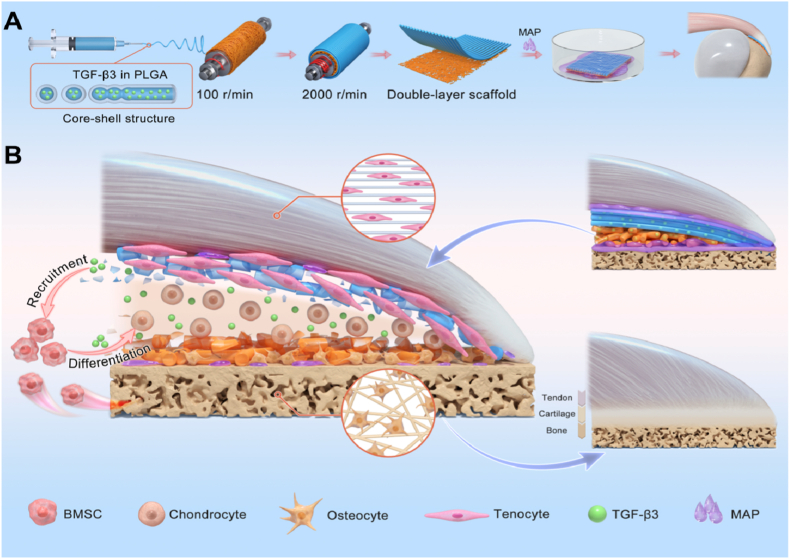
Summary of fabricating a biomimetic double-layered electrospun nanofibrous scaffold with MAP coating and TGF-β3 encapsulation for promoting tendon-bone healing of RCT. (A) The detailed method and process of fabricating composite electrospun nanofibrous scaffold. (B) The mechanism of the tendon-bone interfacial regeneration with the scaffold.

In the present study, we developed a TGF-β3-loaded electrospun nanofibrous scaffold with a biomimetic double-layered structure and MAP coating to enhance tendon-bone healing (Scheme 1), with the objective of restoring the enthesis structure of the rotator cuff. The double-layered structure, featuring dissimilar fibrous arrangements in each layer, was designed to simultaneously mimic the distinct ECM structures of tendon and bone. This design promoted a favorable match with the tendon-bone interface, providing a biomimetic ECM for the ingrowth of multiple tissues. Simultaneously, the MAP-coated scaffold demonstrated superior biocompatibility and adhesion, acting as an intermediate linker that bound the tendon and bone closely, facilitating the establishment of tendon-bone connection in the early stages. Through the sustained release of TGF-β3, the recruitment and induction of stem cells occurred, fostering chondrogenesis and resulting in the formation of a cartilage transition zone at the interface. This process ultimately contributed to the reconstruction of the enthesis structure. To further assess the feasibility and effectiveness of this functionalized electrospun nanofibrous scaffold, we conducted in vitro evaluations, including characterizations of morphology, hydrophilicity, adhesion, TGF-β3 release behavior, and chondro-inductive ability. Additionally, RNA-sequencing was performed to clarify the underlying regulatory mechanism of the composite scaffold. Finally, the scaffold was implanted into the tendon-bone interface in a rat RCT model, and its impact on improving tendon-bone healing was evaluated in vivo.

## 2 Materials and methods

### 2.1 Fabrication of electrospun nanofibrous scaffolds

Four types of electrospun scaffolds were prepared, namely P-PL (phosphate-buffered saline, PBS @ PLGA), T-PL (TGF-β3 @ PLGA), M-PL (MAP @ PLGA), and T-M-PL (TGF-β3 and MAP @ PLGA). For samples containing TGF-β3, initially, 100 μL of TGF-β3 (50 μg/mL, PeproTech, USA) was mixed with 50 μL of hyaluronic acid (HA, Macklin, China) hydrosol (1.2 %, w/w) to form HA-TGF-β3 hydrosol. For the P-PL and M-PL groups, TGF-β3 was replaced with equal volumes of PBS. Subsequently, the hydrosol was added dropwise into a solvent mixture containing 4 mg dichloromethane (DCM, Qiangshun, China) and 0.02 g Span-80 (Sigma-Aldrich, USA). It was then stirred at high speed at room temperature to form a water-in-oil (W/O) emulsion. Following this, 1 g PLGA (65/35, MW = 100 kDa, Daigang, China) and 2 g dimethylformamide (DMF, Qiangsheng, China) were added to the emulsion. The electrospinning process was followed established protocols in the literature [41,42]. Briefly, the prepared solution was loaded into a 5-mL syringe connected to an injection pump (LSP01-3A, Longer Pump, China) at a flow rate of 0.8 mL/h. The needle tip is fixed with a metal clip and connected to a high-voltage power supply (DW-P203-1ACF0, Dong Wen High Voltage, China). The voltage was set to 15 kV, and the distance between the tip and the collector (SIBEINING, China) was 15 cm. The drum collector's rotation speed was set to 2000 rpm for 3 h to fabricate the layer of aligned fibers. Subsequently, the rotation speed was reduced to 100 rpm for another 3 h to fabricate the layer of random fibers on the same collector, resulting in a double-layer structured scaffold. Two milligrams of phenol red were added to the second half of the electrospinning work solution to label the random layer. For MAP working solution, 3.0 mL was prepared by adding 100 μL of MAP (1.5 mg/mL, in 5% acetic acid, Corning, USA) to a neutral buffer solution containing 2.85 mL of 0.1 M sodium bicarbonate and 50 μL of 1 N sodium hydroxide. In the M-PL and T-M-PL groups, the MAP working solution was applied to the scaffolds (70 μL/cm^2^) as a liquid film and incubated for at least 30 min. After washing with sterile water to remove the residual solution, a MAP coating was left. Subsequently, the scaffolds were vacuum-dried for 24 h and stored at 2-8 °C before further use.

### 2.2 Characterization of scaffolds

The morphology of various layers and the cross-section of electrospun scaffolds were examined using scanning electron microscopy (SEM, Quanta 250, FEI, USA) after sputter-coating the scaffolds with gold. The average diameter of nanofibers was measured and analyzed utilizing Image J software (NIH, Bethesda, USA). Additionally, the structure of individual fibers within the scaffold was scrutinized using transmission electron microscopy (TEM, FEI, USA). The molecular structure of scaffolds was detected by Fourier Transform Infrared Spectroscopy (FTIR, Nicolet 6700, Thermo Fisher Scientific, USA). Water contact angles (WCA) were determined using the sessile drop method with a video-enabled goniometer (DSA25, KRUSS, Germany) to assess the hydrophilicity of the scaffolds. The adhesion of the scaffold was evaluated through lap shear tests with glass slides, utilizing a universal mechanical testing system (HY-0580, Hengyi, China) at a speed of 5 mm/min.

### 2.3 TGF-β3 loading and release assay

To confirm the loading and distribution of the TGF-β3 in the scaffold, Cy5 fluorescently-tagged bovine serum albumin (BSA-Cy5, Bioss, China) was utilized as a surrogate for TGF-β3 and added to the electrospun working solution. Electrospun fibers were collected on a glass slide and observed under both bright and fluorescent fields using a fluorescence microscope (Zeiss Axiovert 200, USA).

To investigate the release profile of TGF-β3, electrospun scaffolds weighing 50 mg, theoretically containing 0.25 μg TGF-β3, were immersed in 4 mL PBS with 1% BSA and kept shaking. At specific time points (1, 2, 3, 5, 7, 10, 15, 20, 25, and 30 days), 1 mL of supernatant was collected, and an equal amount of PBS was added. The released TGF-β3 in the supernatant was quantified using a TGF-β3 ELISA kit (Solarbio, China), and the release curve was plotted.

### 2.4 Cell viability and proliferation on scaffolds

All animal experiments strictly adhered to the NIH Guide for the Care and Use of Laboratory Animals and received approval from the Ethics Committee of Soochow University (ECSU). Bone marrow stem cells (BMSCs) were extracted from the femurs of Sprague-Dawley (SD) rats and cultured in α-MEM complete medium (HyClone, USA) supplemented with 10% fetal bovine serum and 1% penicillin-streptomycin (both from Gibco, USA). All experiments were conducted with BMSCs at passage 3. The electrospun scaffolds were customized to encase circular coverslips (14 mm in diameter) with different layers and were subsequently sterilized for use in cellular experiments. BMSCs were seeded at a density of 1 × 10^4^ cells/mL on distinct layers of different scaffolds in 24-well plates. The control group did not contain any scaffold. After culturing for 3 days, live/dead staining was performed to assess the viability of cells on the scaffolds. After removing the medium, cells were incubated with a live/dead staining solution (Invitrogen, USA) at room temperature for 30 min. Images were captured using a fluorescence microscope, and the numbers of live cells per field were counted in three randomly selected visual fields. To evaluate cell proliferation, BMSCs were seeded on scaffolds and cultured for 1, 3, and 5 days. At each time point, cells were incubated in a fresh complete medium containing 10% CCK-8 solution (Dojindo, Japan) for 2 h. Subsequently, 100 μL of the mixed solution was pipetted into a 96-well plate, and absorbance was measured using a microplate reader (BioTek, USA) at a wavelength of 450 nm.

### 2.5 Immunofluorescence

After culturing on different scaffolds with complete medium for 3 days, BMSCs were fixed using 4% paraformaldehyde (Biosharp, China) and subsequently permeabilized with 0.3% Triton X-100 (Sigma-Aldrich, USA) in PBS for 20 min. Following this, cells were blocked with Immunol Staining Block (Beyotime, China) for 1 h. Cells were then incubated with a 1:100 diluted anti-Vinculin primary antibody at 4 °C overnight. Further staining was conducted with Alexa Fluor 488 secondary antibodies (diluted at 1:400, Abcam, UK) at room temperature for 1 h. Subsequently, cells were stained with Rhodamin-Phalloidin (diluted at 1:300, Yeasen, China) for 1 h and DAPI for 10 min. Images were then obtained under a fluorescence microscope.

Furthermore, BMSCs were cultured on different scaffolds with basic chondrogenic differentiation medium comprising high-glucose DMEM, 100 nM dexamethasone, 50 mg/mL ascorbic acid 2-phosphate, 1 mM sodium pyruvate, 40 μg/mL proline (all from Sigma-Aldrich, USA), and 1% insulin transferrin selenium (ITS, Becton Dickinson, USA). After 7 days of culture, collagen Ⅱ (COL Ⅱ) immunofluorescence (anti-COL Ⅱ primary antibody diluted at 1:200) and photography were performed as mentioned above. All the primary antibodies used are listed in Table S1.

### 2.6 In vitro BMSCs recruitment effect

The recruitment effect of scaffolds on BMSCs was assessed through a Transwell assay. In brief, 200 μL of a suspension containing a total of 2 × 10^4^ BMSCs was placed in the upper chamber of a 24-well Transwell plate (8-μm pore size, Corning, USA). Scaffolds were positioned in the lower chamber with 500 μL of medium. Meanwhile, a control group (without scaffold or bioactive factors in the lower chamber) and a TGF-β3 group (without scaffold but with 10 ng/mL TGF-β3 in the lower chamber) were established for comparison with the scaffold groups. Following a 12-h and 24-h incubation period, the Transwell plates were rinsed with PBS and subsequently fixed in 4% paraformaldehyde. Subsequently, cells on the upper side of the Transwell membranes were wiped up with a cotton swab, and then the Transwell membranes were stained with a 0.1% crystal violet solution. Afterwards, the stained cells migrated to the lower chamber were captured and quantified in three randomly selected microscopic fields.

### 2.7 Real-time quantitative PCR (RT-qPCR) analysis

To assess the chondrogenic differentiation of BMSCs cultured on various scaffolds, the expression levels of chondrogenesis-related genes (*Col2a1 and Acan*) in BMSCs were determined using RT-qPCR after incubation in basic chondrogenic differentiation medium for 3 days. Briefly, RNA was extracted from BMSCs using Trizol reagent (Invitrogen, Carlsbad, USA), and the RNA concentration was determined using a NanoDrop 2000 spectrophotometer (Thermo Fisher Scientific, USA). Subsequently, RNA was converted to cDNA using RT Master Mix (ABM, Canada), and RT-qPCR was conducted with SYBR Green Supermix (BioRad, USA) on an Applied Biosystems 7500 Real-Time PCR System (Applied Biosystems, USA). The relative gene expression was calculated using the 2^−ΔΔCt^ method and normalized to *Gapdh*. The primer sequences (Sangon Biotech, China) for the genes utilized in this study are listed in Table S2.

### 2.8 RNA-seq and bioinformatic analyses

To delve into the underlying regulatory mechanism of the composite scaffold on BMSCs, RNA sequencing was performed. After a 3-day culturing, total RNA was extracted from BMSCs in the control and T-M-PL groups using Trizol reagent. Three parallel samples were set in each group. The quantity and purity of each RNA sample were determined using NanoDrop ND-2000, and the RNA integrity was assessed using the Bioanalyzer 2100 (Agilent, USA). High-quality RNA samples (total RNA amount >1 μg, RNA concentration >50 ng/μL, OD value 260/280 range 1.8-2.2, and RIN value > 7.0) were filtrated to construct a sequencing library. The process of library construction involved mRNA purification, capturing, fragmentation, and reverse transcription to cDNA, followed by A-tailing, adaptor ligation, and PCR amplification. Subsequently, RNA-seq was carried out using the Illumina NovaseqTM 6000 (LC-Bio, China). The identification of differentially expressed genes (DEGs) was performed using the edgeR software package (Bioconductor, version 3.32.1, USA) with criteria set at a fold change ≥2 and *p* < 0.05. Finally, Gene Ontology (GO) and Kyoto Encyclopedia of Genes and Genomes (KEGG) enrichment analyses were conducted on the DEGs to gain insights into the biological processes and pathways influenced by the composite scaffold.

### 2.9 Western blot analysis

To further validate the involvement of the regulatory signaling pathway, Western blot analysis was conducted. After 5 days of culture, proteins from BMSCs in the control, P-PL, T-M-PL and T-M-PL + MK-2206 groups were extracted using RIPA lysed buffer (Beyotime, China). The use of MK-2206 was based on a previous study [43], in which BMSCs were treated with MK-2206 (5 μmol/L) as an Akt inhibitor. Protein concentrations in all samples were determined using a BCA Protein Assay Kit (Beyotime, China). Following the separation by 10% SDS-PAGE gels, the proteins were transferred to nitrocellulose membranes (Beyotime, China). The membranes were then blocked for 1 h and incubated with primary antibodies (1:10000 diluted anti-AKT, 1:5000 diluted anti-AKT1/P-AKT, 1:1000 diluted anti-Smad2/3, 1:1000 diluted anti-COL Ⅱ, and 1:10000 diluted anti-GAPDH) at 4 °C overnight. After washing with PBS containing 0.1% Tween (PBST), the membranes were further incubated with 1:1000 diluted peroxidase-conjugated secondary antibodies (Beyotime, China) at room temperature for 1 h. Finally, the immunoreactive bands were visualized using a FluorChem imaging system (Protein Simple, USA). The primary antibodies used in this experiment are listed in Table S1.

### 2.10 Animal model and implantation of scaffolds

Male SD rats aged 12 weeks and weighing about 400 g were procured for the experiment. Five groups were designated in the animal experiments: (ⅰ) defect group with no scaffold implant, (ⅱ) P-PL scaffold group, (ⅲ) T-PL scaffold group, (ⅳ) M-PL scaffold group, and (ⅴ) T-M-PL scaffold group (n = 12 per group). After an intraperitoneal injection of pentobarbital for anesthesia and routine disinfection, a longitudinal incision anterolateral to the midline of the shoulder was made. The deltoid muscle and acromioclavicular joint were separated sequentially, and then rotator cuff tendons were exposed (Fig. S1). Subsequently, the supraspinatus tendon was dissected at its insertion site on the surface of the humeral great tuberosity. Following decortication at the insertion site region, the scaffold was placed between the supraspinatus tendon and the great tuberosity, with the aligned layer (white side of the scaffold) facing upward and parallel to the tendon fiber direction. Afterwards, the detached supraspinatus tendon, along with the scaffold, was reattached to the insertion site through a transosseous suturing with 3-0 Ethibond sutures (Ethicon, USA). The wound was then closed layer by layer, and incisions were disinfected with iodine for three consecutive days postoperatively.

### 2.11 In vivo recruitment effect on stem cells

The in vivo recruitment effect of scaffolds on stem cells was assessed through immunofluorescence of SOX2, a molecular marker for stem cells, including BMSCs. Specimens were harvested 1 week after surgery and fixed in a 10% formaldehyde solution for 24 h. After decalcification with 14% ethylenediaminetetraacetic acid (EDTA) for 30 days, specimens were embedded in paraffin and sliced to a thickness of 6 μm. Immunofluorescence of the slices followed a procedure similar to that described for cells previously. Briefly, after antigen retrieval, the slices were blocked for 1 h and then incubated with a 1:100 diluted anti-SOX2 primary antibody (Table S1) at 4 °C overnight. This was followed by incubation with a Cy3-conjugated secondary antibody (diluted at 1:200, Abcam, UK) at room temperature for 1 h. After nuclei staining with DAPI, sections were observed using a fluorescence microscope. The average number of SOX2 positive cells in interface region was measured and analyzed utilizing Image J software.

### 2.12 Histological analysis

Specimens of supraspinatus tendon-humerus complexes were collected at 4 and 8 weeks post-surgery, followed by fixation, decalcification, paraffin embedding, and slicing, as previously described. H&E staining was performed to observe tissue histology. Safranin O/Fast green (SO/FG) staining was employed to assess the formation of glycosaminoglycan, a major component of the cartilage ECM at the tendon-bone interface. Masson trichrome (MT) staining was utilized to evaluate collagen formation, a crucial indicator of tendon maturity at the insertion site [44]. To analyze changes in the expression of COL Ⅱ protein in vivo, immunohistochemical (IHC) staining was conducted. After antigen retrieval, samples were blocked for 1 h at room temperature. Subsequently, the samples were incubated with a 1:200 diluted anti-COL Ⅱ primary antibody (Table S1) at 4 °C overnight, followed by incubation with a 1:100 diluted HRP-labeled secondary antibody (Beyotime, China) at room temperature for 1 h. Samples were then stained with 3,3′-diaminobenzidine (DAB, Sigma-Aldrich, USA) and hematoxylin sequentially. Finally, sections were photographed using a bright field microscope. The modified tendon maturing score [45] was introduced to evaluate regenerated tendon and tendon-bone insertion. Quantitative analysis of MT staining, SO/FG staining and COL II IHC positively stained area proportion/intensity at the tendon-to-bone interface was performed using Image J software.

### 2.13 Biomechanical tests

At 8 weeks post-surgery, specimens of supraspinatus tendon-humerus complexes were collected. The cross-sectional area of the supraspinatus tendon insertion site on the humerus head was measured using a digital caliper (Fig. 8B). Biomechanical tests were carried out employing an electronic universal mechanics testing machine (TY-8000A, TYTESTER, China) (Fig. 8A). The tendon and humerus were secured with clips and aligned in a straight force line. The specimen was preloaded to 1 N and then loaded to failure at the rate of 10 mm/min. The ultimate load-to-failure was recorded as the maximum force sustained by the humeral bone-supraspinatus tendon complex, and the elongation was also recorded. The stress and stiffness were subsequently calculated as the ultimate load to failure divided by the cross-sectional area or the elongation, respectively.

### 2.14 Statistical analysis

All values were expressed as means ± SD. Statistical analyses were performed using GraphPad Prism software (version 8, USA). One-way analysis of variance (ANOVA) followed by Tukey's test was employed to compare differences among multiple groups. A *p*-value less than 0.05 was considered statistically significant.

## 3 Results

### 3.1 Characterization of scaffolds

**Fig. 1:**
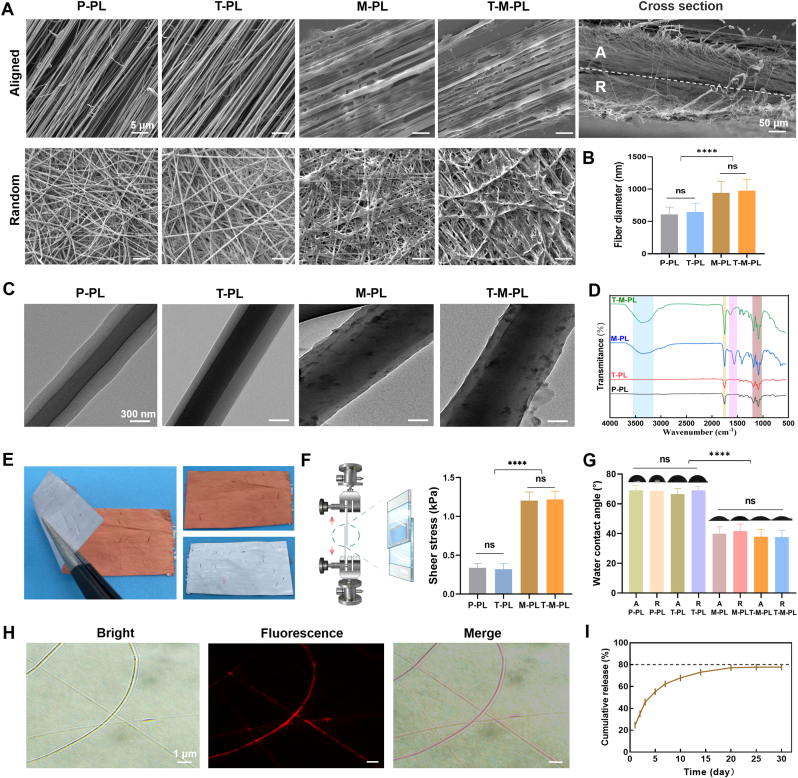
Characterizations of scaffolds. (A) Scanning electron microscopy (SEM) observation of fibrous arrangement in different layers in scaffolds. (B) Diameter of fibers in different scaffolds (n = 50 from three images). (C) Transmission electron microscopy (TEM) images of single fiber structure. (D) Fourier Transform Infrared Spectroscopy (FTIR) spectra of the scaffolds. (E) Macroscopic observation of double-layered scaffold. The random layer was labeled with phenol red. (F) Lap shear test to evaluate the adhesion of scaffolds. (G) Water contact angles (WCA) of different scaffolds. (H) The distribution of BSA-Cy5 in the fibers. (I) In vitro release kinetics of TGF-β3 in T-M-PL scaffold. (A, aligned layer; R, random layer. P, PBS; T, TGF-β3; M, mussel adhesive protein (MAP); PL, PLGA. ∗∗∗∗*p* < 0.0001; ns, no significant difference).

SEM analysis was conducted to observe the fibrous arrangement in each layer of the four kinds of scaffolds (P-PL, T-PL, M-PL, and T-M-PL). Fibers exhibited a parallel arrangement in aligned layer and an irregular orientation in random layer (Fig. 1A), mimicking ECM structures of tendon and bone respectively. The mean diameters of the fibers in four scaffolds were 607.6 ± 114.3 nm (P-PL), 648.4 ± 133.4 nm (T-PL), 943.6 ± 178.7 nm (M-PL), and 977.1 ± 173.6 nm (T-M-PL), respectively (Fig. 1B and S2). There was a significant difference between the MAP-coated groups and non-MAP groups. No substantial difference in fiber diameter was observed between groups with or without TGF-β3. All fibers displayed a smooth surface, and minimal beading was observed in the visual fields. TEM images confirmed the core-shell fibrous microstructure in all groups, with the MAP coating wrapping the nanofiber in the M-PL and T-M-PL groups (Fig. 1C). FTIR spectra (Fig. 1D) exhibited the characteristic ester carbonyl absorption (1750 cm^−1^) and C–O–C/C–O absorptions (1000–1200 cm^−1^) of PLGA. M-PL and T-M-PL scaffolds showed characteristic amide absorption (1540–1660 cm^−1^) and N–H/O–H absorptions (3200–3600 cm^−1^) of MPA, suggesting the successful assembly of MAP to the scaffold. The macroscopic observation of the scaffold is shown in Fig. 1E, where the random layer was labeled with phenol red, and the aligned layer was represented by the white side of the scaffold. Additionally, the lap shear test with glass slides was employed to assess scaffold adhesion, revealing a significantly higher shear stress in groups with MAP than those without MAP (Fig. 1F). Furthermore, the hydrophilicity of each layer in different kinds of scaffolds was determined through the sessile drop experiment. As illustrated in Fig. 1G, the WCA of MAP-coated scaffolds was significantly lower than that in non-MAP groups, indicating superior scaffold hydrophilicity due to MAP coating. Meanwhile, no apparent difference in WCA was observed between groups with or without TGF-β3, suggesting that TGF-β3 did not affect scaffold hydrophilicity.

### 3.2 TGF-β3 distribution and release

Fig. 1H illustrates the distribution of fluorescent BSA-Cy5 in the nanofibers, indicating successful cytokine loading in the scaffold through the emulsion electrospinning technique. Fig. 1I presents the release behavior of TGF-β3 from the T-M-PL scaffold and the cumulative release (%) were 24.68 ± 1.03 (1 d), 45.83 ± 0.24 (3 d), 55.33 ± 0.41 (5 d), 67.84 ± 0.45 (10 d), 73.09 ± 0.13 (15 d), 77.10 ± 0.06 (20 d), and 77.75 ± 0.11 (30 d). An initial burst release was observed in the first 3 days, followed by a stable and sustained release for more than 20 days. The cumulative release of TGF-β3 reached up to nearly 80% over 30 days and the entire release process conforms to the Korsmeyer-Peppas release kinetic model (Fig. S3).

### 3.3 Cell viability, morphology, adhesion, and proliferation on scaffolds

**Fig. 2:**
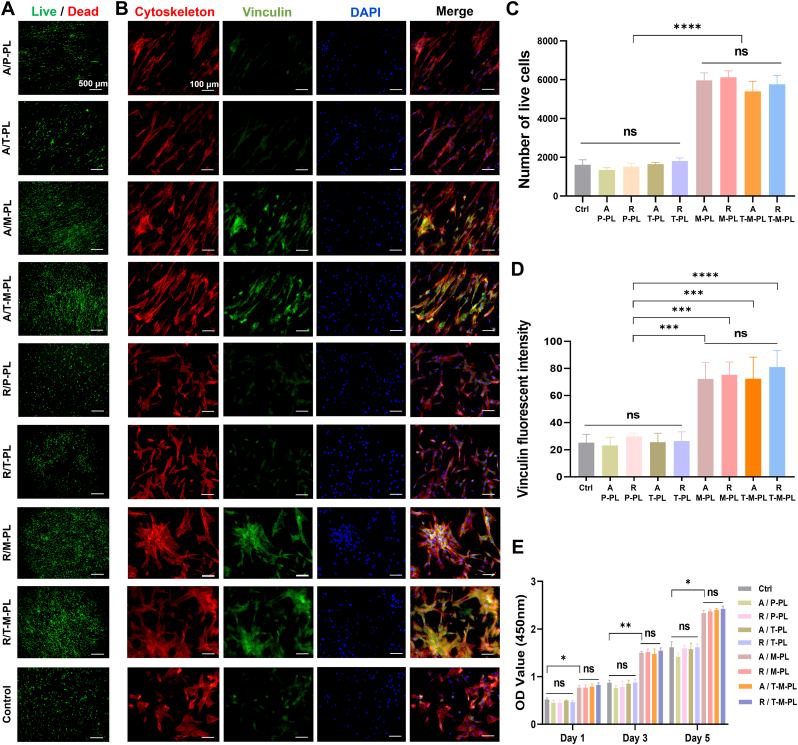
Cell viability, morphology, adhesion, and proliferation on different layers of each scaffold. (A) Live/dead staining. (B) Cytoskeleton, nuclei, and Vinculin immunofluorescence. (C) Number of live cells in live/dead staining of different groups. (D) Vinculin fluorescent intensity of different groups. (E) CCK-8 cell proliferation assay. (A/, aligned layer; R/, random layer. P, PBS; T, TGF-β3; M, mussel adhesive protein (MAP); PL, PLGA; OD, Optical Density. ∗*p*< 0.05; ∗∗*p*< 0.01; ∗∗∗*p*< 0.001; ∗∗∗∗*p* < 0.0001; ns, no significant difference.).

The assessment of cell viability on scaffolds was conducted through live/dead staining. As shown in Fig. 2A, numerous live cells with few dead cells were observed in all groups, demonstrating the nontoxicity of PLGA electrospun scaffolds. Additionally, more live cells were observed in groups with MAP, indicating that MAP coating conferred superior biocompatibility to the scaffolds. No significant differences were noted on the aligned or random layers of the same kind of scaffold and in groups with or without TGF-β3 (Fig. 2C), suggesting that fibrous arrangement and TGF-β3 did not affect biocompatibility of the scaffold. Cytoskeleton staining (Fig. 2B, first column and Fig. S4) revealed distinct morphologies and arrangements of BMSCs on different layers of scaffolds. On the aligned layer of each scaffold, BMSCs appeared elongated and highly ordered in accordance with the direction of fiber alignment, resembling tendon cells in native tendon tissue. On the random layer, BMSCs exhibited irregular shapes and random orientations, akin to those in native bone tissue. Vinculin immunofluorescence showed significantly higher fluorescence intensity in groups with MAP than those without MAP (Fig. 2B, second column, and D), indicating that MAP coating improved cell adhesion on the scaffolds. Meanwhile, no apparent difference in fluorescence intensity was observed among groups without MAP, indicating that fiber arrangement and TGF-β3 did not significantly affect cell adhesion on the scaffolds. Moreover, the proliferation of BMSCs on scaffolds was evaluated using a CCK-8 assay. On days 1, 3, and 5 after culturing, cell proliferation was notably higher in groups with MAP than those without MAP (Fig. 2E), suggesting that the MAP coating promoted cell proliferation on the scaffold. No significant differences were observed between groups with or without TGF-β3, indicating that TGF-β3 had no discernible effect on the proliferation of BMSCs.

### 3.4 The effect on BMSC recruitment and chondrogenic differentiation in vitro

**Fig. 3:**
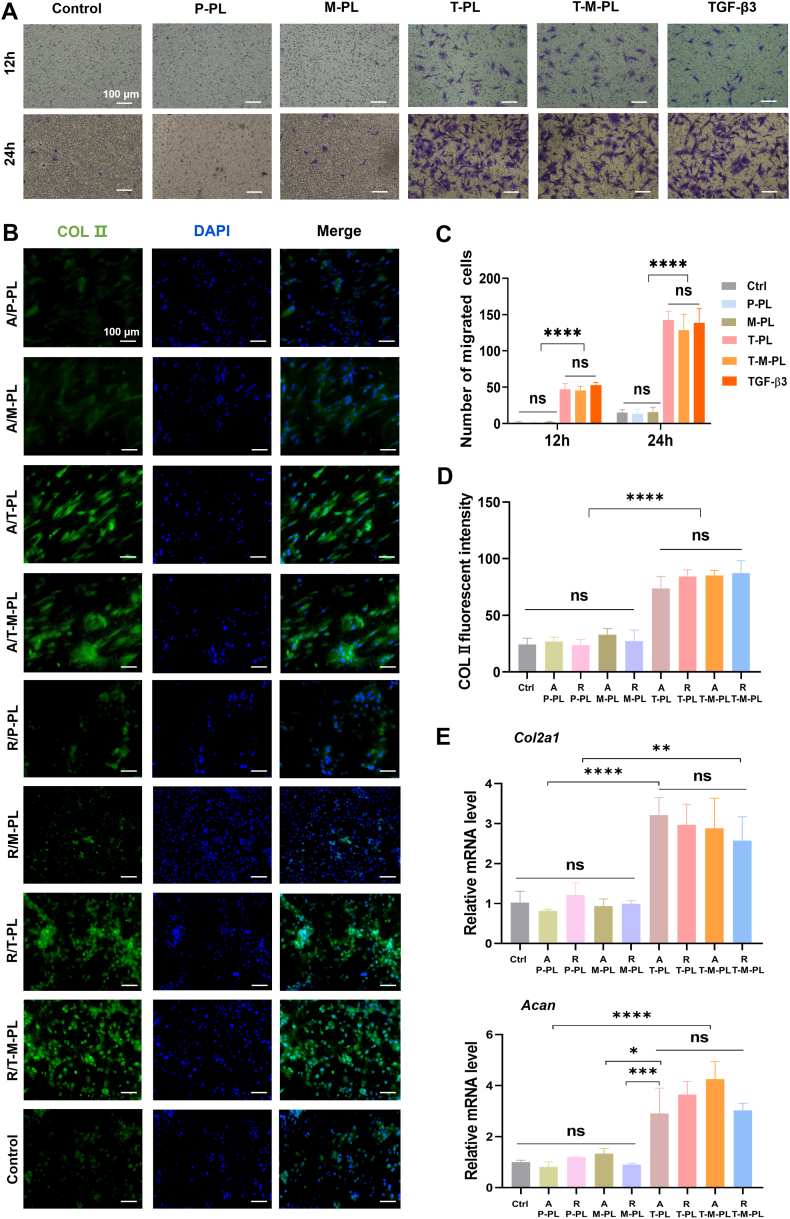
In vitro recruitment and pro-chondrogenic differentiation effect of scaffolds on BMSCs. (A) Representative images of migrated BMSCs in Transwell experiment at 12 and 24 h. (B) COL Ⅱ immunofluorescence of BMSCs cultured on different layers of each scaffold. (C) Quantitative analysis of migrated cells. (D) COL Ⅱ fluorescent intensity of different groups. (E) Quantifications of chondrogenesis-related gene expression (*Col2a1* and *Acan*) in BMSCs on different scaffolds by RT-qPCR. (Control group: no scaffolds or bioactive factors. A/, aligned layer; R/, random layer. P, PBS; T, TGF-β3; M, mussel adhesive protein (MAP); PL, PLGA. ∗*p*< 0.05; ∗∗*p*< 0.01; ∗∗∗*p*< 0.001; ∗∗∗∗*p* < 0.0001; ns, no significant difference.).

The recruitment effect of scaffolds on BMSCs was investigated through the Transwell experiment. Cell migration was observed in groups with TGF-β3, while nearly no cells migrated to the lower chamber in groups without TGF-β3 at 12 h (Fig. 3A, first row). After 24 h of culture, the number of cells migrated to the lower chamber was significantly larger in groups with TGF-β3 (Fig. 3A second row and C). The TGF-β3-loaded scaffold groups (T-PL and T-M-PL) exhibited equivalent migrated cell number to that of 10 ng/mL TGF-β3 group, confirming the recruitment effect of the TGF-β3 release from scaffolds on BMSCs. The expression of the chondrogenesis-related protein COL Ⅱ in BMSCs was assessed through immunofluorescence. As shown in Fig. 3B and D, the fluorescent intensity representing the expression level of COL Ⅱ was markedly higher in groups with TGF-β3 than those without TGF-β3, indicating that TGF-β3 released from the scaffolds effectively induced the chondrogenic differentiation of BMSCs. Furthermore, no apparent differences were detected in the aligned or random layers of the same kind of scaffold, demonstrating that the chondrogenic differentiation of BMSCs was not associated with the fiber arrangement of the scaffold. Additionally, chondrogenesis-related gene expression was quantified using RT-qPCR. The expression levels of both *Col2a1* and *Acan* were significantly higher in groups with TGF-β3 (Fig. 3E), consistent with the immunofluorescence results.

### 3.5 Mechanism underlying the regulatory effect of composite scaffold on BMSCs

**Fig. 4:**
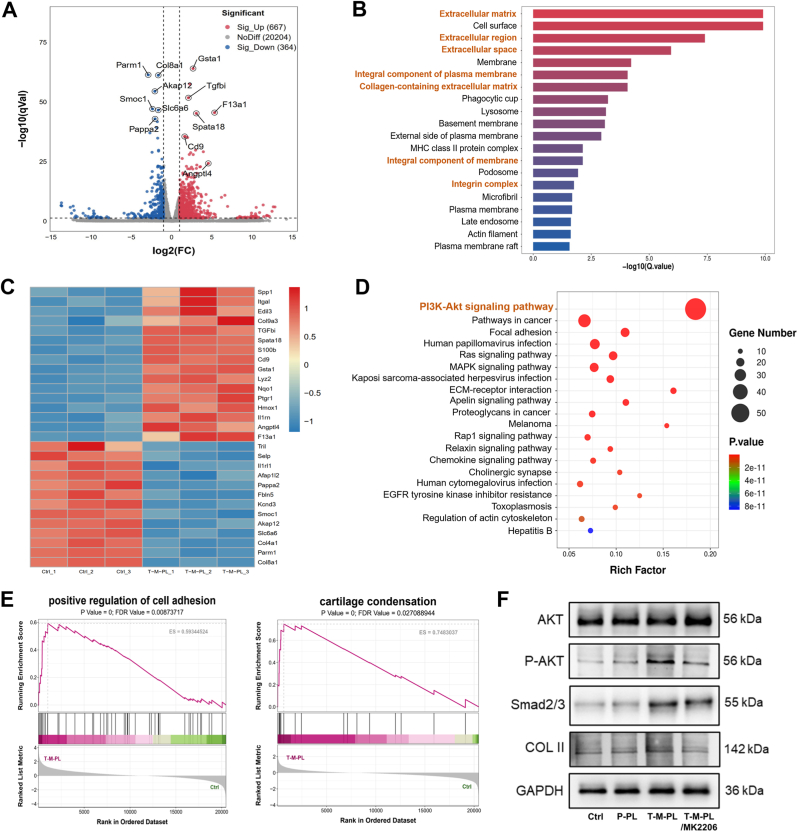
RNA-seq, bioinformatic, and mechanism analyses for the regulatory effect of composite scaffold on BMSCs. (A) Volcano plot of differentially expressed genes (DEGs). (B) Gene Ontology (GO) analysis. (C) Gene expression heatmap. (D) Kyoto Encyclopedia of Genes and Genomes (KEGG) enrichment. (E) Gene set enrichment analysis (GSEA). (F) Western blot analysis. (P, PBS; T, TGF-β3; M, mussel adhesive protein (MAP); PL, PLGA; P-AKT, phosphorylated AKT).

To further clarify the underlying mechanisms responsible for the regulatory effect of the composite scaffold on BMSCs, we conducted RNA-seq. Principal components analysis (Fig. S5) revealed a distinct transcriptome profile between the control group (Ctrl, no scaffold or bioactive factors) and the T-M-PL group, with low dispersion observed among the three duplicated samples in each group. The volcano plot (Fig. 4A) showed that 667 genes were significantly upregulated, and 364 genes were downregulated in BMSCs cultured on the T-M-PL scaffold compared with the control group. The gene expression heatmap displayed a subset of DEGs between the two groups (Fig. 4C). Genes related to cell adhesion (*Spp1*, *Itga1*, *Edil3*, etc.) and ECM (*Tgfbi*, *Col9a3*, *Angptl4*, etc.) were upregulated in the T-M-PL group compared with the control group. To unveil the functions of highly expressed genes in the T-M-PL group, GO analysis was performed. As shown in Fig. 4B, the DEGs between the two groups were enriched in GO terms including ECM, collagen-containing ECM, cell surface, and integrin complex. Consistently, gene set enrichment analysis (GSEA) indicated that DEGs associated with the positive regulation of cell adhesion and cartilage condensation were upregulated in the T-M-PL group (Fig. 4E), confirming the promoting effects of the composite scaffold on cell adhesion and ECM formation. To clarify the potential signaling pathways involved, a KEGG pathway enrichment analysis was performed. As shown in Fig. 4D, the PI3K-Akt signaling pathway exhibited the largest number of enriched DEGs, suggesting that the functions of the composite scaffolds were likely regulated by the PI3K-Akt signaling pathway. The Western blot analysis revealed that phosphorylated AKT (P-AKT) and COL Ⅱ expression levels were simultaneously and the most prominently upregulated in the T-M-PL group (Fig. 4F). To further verify the involvement of Akt signaling, MK-2206 was introduced as a pharmacological inhibitor. The results showed that inhibition of Akt phosphorylation significantly reduced the expression of P-AKT and COL II in the T-M-PL + MK-2206 group, whereas total AKT expression showed no obvious change. Furthermore, the key protein Smad2/3 in the TGF-β-Smad signaling pathway was upregulated in the T-M-PL group and unchanged by MK-2206 treatment. These findings provide pharmacological evidence that the composite scaffold as a whole promoted collagen-containing ECM formation, mainly through PI3K-Akt signaling pathway.

### 3.6 Performance of scaffolds in vivo

#### 3.6.1 In vivo recruitment effect on stem cells

**Fig. 5:**
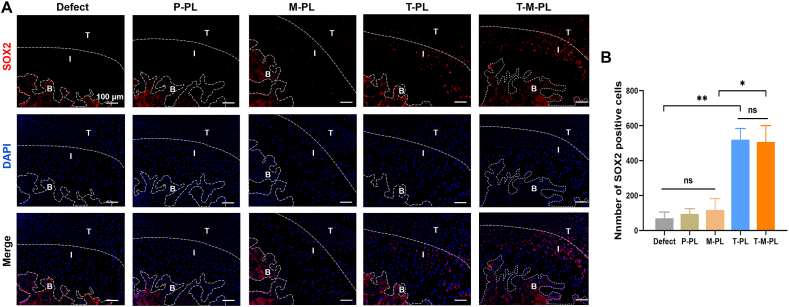
Stem cell recruitment effect in vivo. (A) SOX2 immunofluorescence. BMSCs were marked with red fluorescence and recruited to the tendon-bone interface in T-PL and T-M-PL groups. (B) Number of SOX2 positive cells. (T-I-B: T, tendon; I, tendon-bone interface; B, bone. P, PBS; T, TGF-β3; M, mussel adhesive protein (MAP); PL, PLGA. ∗*p*< 0.05; ∗∗*p* < 0.01; ns, no significant difference).)

The in vivo recruitment effect on stem cells was assessed through SOX2 immunofluorescence. As shown in Fig. 5A, red fluorescence was observed in bone and bone marrow tissues in all groups, confirming that BMSCs were probably labeled by SOX2 immunofluorescence. Moreover, a larger number of SOX2 positive cells with red fluorescence were observed near the tendon-bone interface in T-PL and T-M-PL groups compared to other groups, indicating that TGF-β3-loaded scaffolds could effectively recruit BMSCs to the interface region.

#### 3.6.2 Histology evaluation

**Fig. 6:**
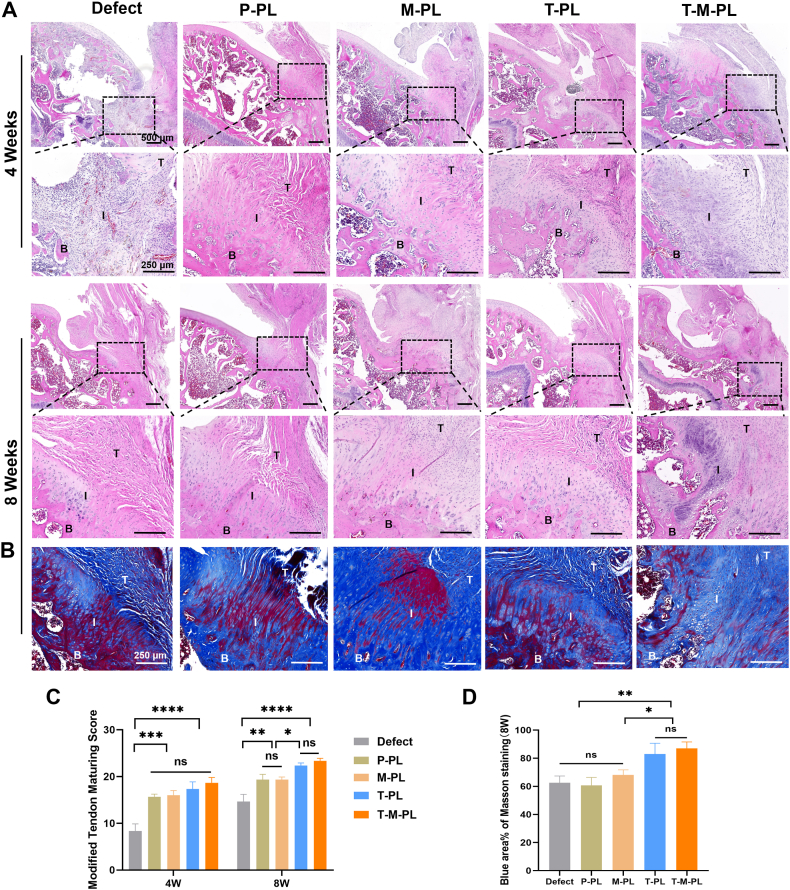
Histological evaluation of the animal experiment. (A) H&E staining at 4 and 8 weeks after surgery. (B) Masson staining at 8 weeks after surgery. (C) Modified tendon mature score. (D) Blue area proportion of Masson staining at 8 weeks after surgery. (T-I-B: T, tendon; I, tendon-bone interface; B, bone. P, PBS; T, TGF-β3; M, mussel adhesive protein (MAP); PL, PLGA. ∗*p*< 0.05; ∗∗*p*< 0.01; ∗∗∗*p*< 0.001; ∗∗∗∗*p* < 0.0001; ns, no significant difference).

**Fig. 7:**
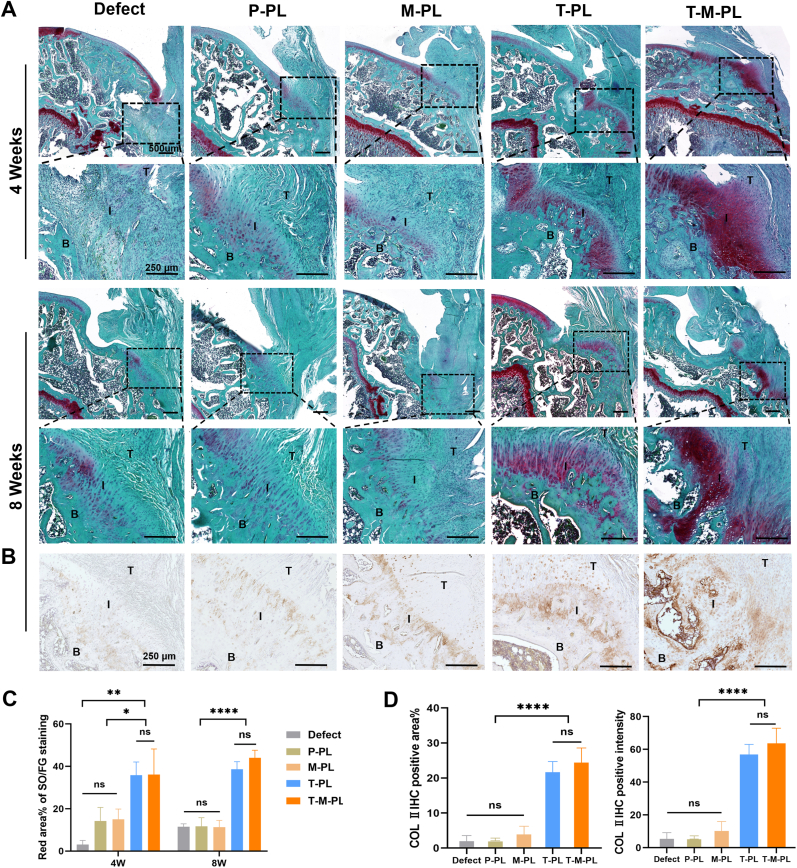
Chondrogenic effect of scaffolds at tendon-bone interface in vivo. (A) Safranin O/Fast green (SO/FG) staining at 4 and 8 weeks after surgery. (B) COL Ⅱ immunohistochemistry (IHC) staining at 8 weeks after surgery. (C) Red area proportion of SO/FG staining. (D) Positive area proportion and intensity of COL Ⅱ IHC staining at 8 weeks after surgery. (T-I-B: T, tendon; I, tendon-bone interface; B, bone. P, PBS; T, TGF-β3; M, mussel adhesive protein (MAP); PL, PLGA. ∗*p*< 0.05; ∗∗*p*< 0.01; ∗∗∗*p*< 0.001; ∗∗∗∗*p* < 0.0001; ns, no significant difference).

H&E staining was employed to examine the gross tissue structure, focusing on cell arrangement and density at the tendon-bone interface. As illustrated in Figs. 6A，at 4 weeks after surgery, the tissue morphology at the interface appeared chaotic in the defect group without scaffold implant. In contrast, groups with scaffold implants exhibited a more organized arrangement of cells and tendon fibers at the interface, with higher modified tendon maturing scores, suggesting that the scaffold played a role in shaping cells and neotissues growing along it. Additionally, neotissues at the interface appeared denser in groups with MAP than those without MAP, indicating the favorable biocompatibility of the MAP-coated scaffolds, which contributed to superior cell adhesion and proliferation. After 8 weeks, more regularly organized and denser tissue was observed in the M-PL and T-M-PL groups. Collagen formation at the tendon-bone interface was evaluated through MT staining. As illustrated in Fig. 6B and D, the defect group showed a small proportion of blue-stained area at the interface, indicating limited collagen formation and inferior tendon maturity. In contrast, the T-M-PL group exhibited the highest level of collagen formation at the interface, with the largest proportion of blue-stained area, suggesting that the composite scaffold effectively promoted collagen formation and tendon maturity at the interface. Furthermore, the formation of glycosaminoglycans was assessed through SO/FG staining. More metachromasia area proportion was observed at the interface in groups with TGF-β3 at both 4 and 8 weeks postoperatively (Fig. 7A and C), indicating that the sustained release of TGF-β3 from scaffolds promoted the formation of glycosaminoglycans, facilitating the regeneration of the cartilage matrix at the tendon-bone interface. COL Ⅱ IHC staining demonstrated the formation level of COL Ⅱ protein. A larger metachromasia area proportion and higher intensity were observed at the interface in groups with TGF-β3 at 8 weeks (Fig. 7B and D), consistent with the SO/FG staining results. The T-M-PL group showed the largest metachromasia zone on both SO/FG staining and COL Ⅱ IHC staining, indicating that the composite scaffold effectively promoted chondrogenesis in vivo at the tendon-bone interface.

**Fig. 8:**
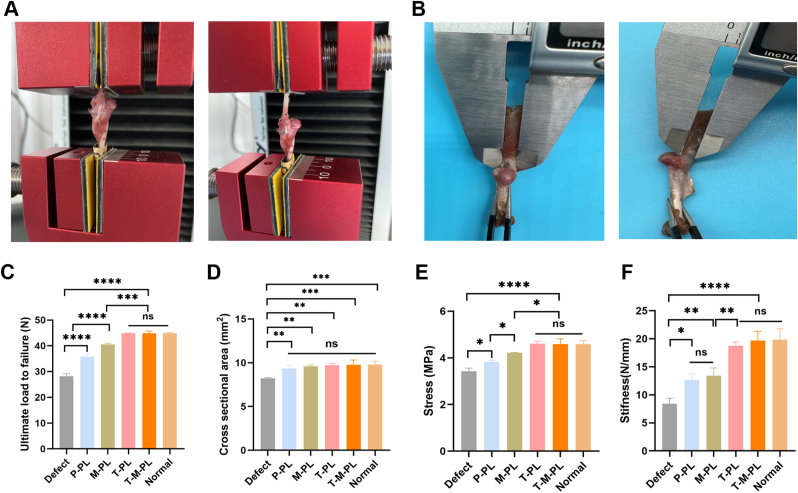
Biomechanical evaluation at 8 weeks postoperatively. (A) Uniaxial tensile test of the humeral bone-supraspinatus tendon complex. (B) Measurement of the tendon insertion cross-sectional area using a digital caliper. (C) Ultimate load to failure of the humeral bone-supraspinatus tendon complex. (D) Cross-sectional areas of the enthesis in different groups. (E) Stress of the humeral bone-supraspinatus tendon complex. (F) Stiffness of the humeral bone-supraspinatus tendon complex. (P, PBS; T, TGF-β3; M, mussel adhesive protein (MAP); PL, PLGA. ∗*p*< 0.05, ∗∗*p*< 0.01, ∗∗∗*p*< 0.001, ∗∗∗∗*p* < 0.0001; ns, no significant difference.).

#### 3.6.3 Biomechanical testing

Biomechanical tests were performed using an electronic universal mechanics testing machine and Fig. 8A shows the representative pre-and-post-test images. During tensile testing, as stretching proceeds, the humerus-tendon complex typically undergoes elongation and deformation in the tendon portion, eventually leading to failure or rupture. The cross-sectional area at the insertion site of the tendon on humerus bone surface (rotator cuff enthesis) was measured using a digital caliper (Fig. 8B). As shown in Fig. 8D, the cross-sectional area of the enthesis was significantly larger in groups with scaffold implants than in the defect group. No significant differences were observed among the groups with scaffold implants and normal specimens, confirming that the scaffold provided sufficient space for neotissue regeneration. The ultimate load-to-failure, stress and stiffness values for the humeral bone-supraspinatus tendon complex of the T-PL and T-M-PL groups at 8 weeks after surgery were higher than other groups and were comparable to normal specimens (Fig. 8C–E and F). This suggested that the cartilage formation induced by the sustained release of TGF-β3 at the tendon-bone interface was critical for restoring the biomechanics of the rotator cuff.

## 4 Discussion

Poor tendon-bone healing is a challenging issue leading a high retear rate after RCT repair [3,5]. Two primary factors contribute to this issue. Firstly, the intricate tendon-bone interface comprises different tissues with distinct structures, making it difficult for them to effectively integrate. Secondly, there is insufficient chondrogenesis to establish a cartilage transitional zone between the tendon and bone, which is crucial for restoring the native enthesis structure of the rotator cuff [5,6,10,[46], [47], [48]]. To meet these challenges, we developed a double-layered biomimetic structure electrospun nanofibrous scaffold encapsulating TGF-β3, with a surface modified by MAP. This innovative approach provided a novel strategy for enhancing tendon-bone healing.

Establishing tissue growth and connection between tendon and bone constitutes a fundamental and crucial step in tendon-bone healing. In this study, we fabricated a MAP-coated electrospun nanofibrous scaffold and implanted it into the tendon-bone interface. The electrospun nanofibrous scaffold served as an ECM, supporting multiple tissue ingrowth and featured favorable adhesion through MAP coating, acting as an intermediate linker binding tendon and bone closely. Biomechanical shear tests demonstrated superior interfacial adhesion force in MAP-coated scaffold groups. Meanwhile, we observed a higher number of viable cells at the early stage and enhanced cell proliferation in the MAP-coated scaffold groups. We attribute this to the improved initial cell adhesion conferred by the MAP coating, which involves the following two mechanisms: (1) From the perspective of scaffold physical properties, PLGA is inherently hydrophobic, limiting cell attachment. The MAP coating reduces scaffold surface hydrophobicity (as shown in Fig. 1G, scaffolds with the MAP coating exhibited lower water contact angles), facilitating initial cell attachment on the scaffold. (2) From the perspective of scaffold biological properties, MAP-coating contains abundant DOPA and adhesive chemical groups, which enhance cell adhesion to the scaffold, as confirmed by vinculin immunofluorescence staining. As a result, the MAP-coated scaffold group had a larger number of viable cells attached to the scaffold at the early stage, leading to correspondingly superior subsequent cell proliferation. Consequently, H&E staining verified denser neotissues and connections between the tendon and bone at the interface in groups with MAP in the early stage. Besides, we noticed that MAP-coated scaffolds (M-PL, T-M-PL) exhibit larger fiber diameters (∼900 nm vs. ∼600 nm) than non-MAP groups (P-PL, T-PL). Whether the change in fiber diameter of scaffolds will affect cell proliferation is concerned. Literature [49,50] has reported that the fiber diameter of the scaffold does affect cell bioactivities and Wang [50] reported that a 400 nm fiber diameter electrospun scaffold is more conducive to cell growth than an 800 nm fiber diameter scaffold. This might be because the smaller fiber diameter scaffold providing larger surface area which is beneficial for cell adhesion and proliferation. However, in this paper we noticed that the CCK-8 assay shows contrary results that the MAP group with larger fiber diameters had more superior cell proliferation. This strongly confirmed from the side that the MAP is the main factor promoting cell proliferation.

Moreover, it is crucial to emphasize that the scaffold structure must be compatible with the structure of adjacent tissues. This is because the scaffold determines the shape and arrangement of the neotissues growing along it, subsequently influencing their functions [14,51,52]. However, the tendon-bone interface comprises both soft and hard tissues with dramatically dissimilar structures. Previous research has reported that collagen fibers, the main components in the ECM acting as the backbone for maintaining tissue structure, exhibit alignment on the tendon side while showing randomness on the bone side of the interface [8,10]. Therefore, conventional scaffolds with a single structure are unable to match the two different tissue structures simultaneously at the tendon-bone interface. In the present study, we fabricated a structurally biomimetic electrospun nanofibrous scaffold with a double-layered structure featuring different fibrous arrangements in each layer. SEM observation showed an orderly and parallel arrangement of fibers in the upper layer, consistent with the ECM structure in tendon tissue, while fibers appeared random in the lower layer, resembling the ECM structure in bone. Cytoskeleton staining demonstrated that cells appeared elongated and oriented in the aligned layer, similar to tendon cells in native tendon tissue. In contrast, cells in the random layer became irregularly shaped and randomly oriented, resembling those in native bone tissue. The different cell morphologies and arrangements on each layer substantiated the structurally biomimetic effect of the double-layered scaffold in vitro. Furthermore, H&E and MT staining showed that tendon fibers were more orderly, with increased collagenesis at the insertion region in groups with scaffold implants compared to those in the defect group, confirming the biomimetic effect of the scaffold in vivo. Consequently, the double-layered electrospun nanofibrous scaffold exhibited a favorable structurally biomimetic property, capable of matching both tendon and bone tissue simultaneously, providing a biomimetic environment for multiple tissue regeneration at the interface.

The formation of a cartilage transition zone at the tendon-bone interface is crucial for reestablishing the native enthesis structure and achieving reliable tendon-bone healing [6,8,9]. Due to the scarcity of chondrocytes and limited intrinsic chondrogenic capacity, there is insufficient cartilage formation at the tendon-bone interface, resulting in scar tissue replacement with inferior biomechanical strength [34,53]. Various exogenous stem cells with cartilage differentiation potential, including BMSCs, have been explored as seed cells for promoting cartilage regeneration in vivo [16,[54], [55], [56], [57]]. However, this approach has drawbacks, including immunological rejection, donor shortage, and low cell survival rates after transplantation. In contrast, recruiting autologous stem cells in situ at the interface through cytokines or drugs can circumvent these issues. In this study, TGF-β3 was loaded into an electrospun nanofibrous scaffold and delivered to the tendon-bone interface to promote in situ recruitment and chondrogenic differentiation of autologous stem cells. Transwell experiments and SOX2 immunofluorescence of tissue sections confirmed the scaffold's capability for stem cell recruitment in vitro and in vivo respectively. It is noteworthy that TGF-β3, being a bioactive protein, may become unstable and ineffective under unfavorable conditions. Moreover, its short active half-life may lead to insufficient concentrations for maintaining biological efficacy. Therefore, a reliable and sustained delivery system is essential. In this study, we employed emulsion electrospinning to encapsulate TGF-β3-HA hydrosol into PLGA nanofibers, forming a core-shell structure unit, as confirmed by TEM observation. HA hydrosol served as a protective agent preventing TGF-β3 denaturation and inactivation during the high-voltage electrospinning process. Compared with surface absorption or chemical coupling methods, the core-shell structure encapsulation provided better storage and protection for TGF-β3. We noticed that ELISA assay showed a rapid TGF-β3 release in the early stage (0-5 days). This might be related to two reasons: (1) the concentration gradient difference of TGF-β3 inside and outside the scaffold, which led to the diffusion of TGF-β3; (2) Some TGF-β3 remaining on the fiber surface without being encapsulated into the fiber interior dissociates and is rapidly released. In the later stage, as the scaffold gradually degraded, the TGF-β3 stored inside the PLGA fibers was continuously released for nearly 30 days, reaching a cumulative volume of nearly 80% in vitro and conforming to Korsmeyer-Peppas release kinetic model. As TGF-β3 was continuously and effectively released, it subsequently promoted the expression of cartilage-related genes. COL Ⅱ immunofluorescence, Western blot analysis, and RT-qPCR demonstrated significantly higher expressions of chondrogenesis-related proteins and genes in groups with TGF-β3, confirming the pro-chondrogenic differentiation effect of TGF-β3-loaded scaffolds in vitro. In vivo experiments, both SO/FG staining and COL Ⅱ IHC staining at 8 weeks showed a larger metachromasia area with higher intensity at the tendon-bone interface in TGF-β3-loaded scaffold groups. The gradient structure of the enthesis was also observed, indicating that the sustained release of TGF-β3 effectively promoted chondrogenesis to form the enthesis structure in vivo. Additionally, biomechanical testing exhibited higher ultimate load-to-failure, stress and stiffness in groups with TGF-β3, similar to normal specimens. These results indicated that by restoring the enthesis structure with a cartilage transition zone, the biomechanical strength of the rotator cuff was regained accordingly.

To further elucidate the underlying regulatory mechanism of the T-M-PL composite scaffold, we investigated the gene expression profile of BMSCs treated with the composite scaffold using RNA-seq. The sequencing data revealed that the DEGs were enriched in GO terms related to the ECM and collagen-containing ECM. The expressions of genes associated with these terms were markedly up-regulated in the T-M-PL group compared with the control group. Additionally, KEGG pathway enrichment analysis demonstrated that the PI3K-Akt signaling pathway was the most relevant. The PI3K-Akt signaling pathway is known to be associated with various cellular biological processes, including cell proliferation, adhesion, migration, differentiation, and ECM regulation [[58], [59], [60]]. Previous studies have reported that activating the PI3K-Akt signaling pathway increases the expression of cartilage-specific proteins such as Col Ⅱ and Aggrecan in nucleus pulposus cells [61]. Conversely, inhibiting the PI3K-Akt signaling pathway upregulates the expression level of MMP13, a protein that facilitates ECM degradation. Moreover, studies have shown that the activation of the PI3K-Akt signaling pathway by bone morphogenetic protein 2 (BMP2) reduces ECM degradation [62]. In our present study, Western blot analysis demonstrated that the expression of both P-AKT and COL Ⅱ was upregulated simultaneously in the T-M-PL groups and downregulated in response to the inhibitor MK-2206, whereas total AKT remained relatively unchanged. Meanwhile, the key protein Smad2/3 in the TGF-β-Smad signaling pathway was upregulated in the T-M-PL group but unchanged with MK-2206 treatment. The expression trend of Smad2/3 is not completely consistent with the changes in Col Ⅱ expression. Therefore, we speculated that the T-M-PL composite scaffold promoted cartilaginous ECM formation possibly mainly through PI3K-Akt signaling pathway. This finding suggested a potential mechanism by which the composite scaffold enhanced the chondrogenic differentiation of BMSCs and contributed to the formation of a functional ECM at the tendon-bone interface.

Several limitations were encountered in this study. Firstly, an acute RCT rat model was established, while some RCT cases in clinical settings are the result of chronic degeneration of the rotator cuff. The acute model might not fully replicate the complex pathological changes associated with chronic degeneration. Secondly, while the favorable biocompatibility and adhesion of the MAP coating were demonstrated, the relatively high cost of MAP could be a potential concern for widespread application, especially in a clinical context. Third, the degradation products of PLGA, lactic acid and glycolic acid, may affect the pH and have adverse effects on cells and tissue regeneration. This issue needs to be addressed and further resolved. Lastly, due to limitations of the experimental equipment, our study lacked further assessments such as magnetic resonance imaging (MRI) to evaluate tendon-bone healing in vivo and gait analysis to evaluate animal activity and behavior. Addressing these limitations in future studies could enhance the translational relevance and applicability of the findings to the clinical context.

## 5 Conclusion

In this study, we successfully developed a TGF-β3-loaded electrospun nanofibrous scaffold featuring a double-layered biomimetic structure and a MAP coating. This innovative scaffold aimed to enhance tendon-bone healing following RCT repair. The MAP coating significantly improved the scaffold's biocompatibility and adhesion, serving as an effective intermediary link between tendon and bone during the early stages of tissue connection. Additionally, the double-layered structure, designed to mimic the respective ECM of tendon and bone in each layer, provided a structurally biomimetic environment conducive to multiple tissue ingrowth. This resulted in cell morphology and behavior that closely mirrored those of adjacent tissues. Furthermore, the sustained release of TGF-β3 from the scaffold played a crucial role in promoting cartilage formation at the tendon-bone interface. This was achieved by inducing stem cell recruitment and chondrogenic differentiation, ultimately restoring the gradient enthesis structure and biomechanical strength of the rotator cuff. In summary, our novel functionalized scaffold demonstrated significant efficacy in promoting tendon-bone healing after RCT repair, presenting a promising strategy for tissue engineering of the tendon-bone interface.

## CRediT authorship contribution statement

**Sheng Fang:** Conceptualization, Data curation, Formal analysis, Funding acquisition, Investigation, Methodology, Writing – original draft. **Yiming Wang:** Formal analysis, Supervision, Visualization, Writing – original draft. **Huan Li:** Conceptualization, Funding acquisition, Investigation, Visualization, Writing – review & editing. **Hanwen Li:** Data curation, Formal analysis, Methodology. **Zhuang Zhu:** Formal analysis, Methodology, Software. **Huan Wang:** Data curation, Investigation, Methodology. **Feng Han:** Formal analysis, Investigation, Methodology. **Shenghao Wang:** Formal analysis, Investigation, Methodology. **Dachuan Liu:** Investigation, Methodology, Visualization. **Jiaying Li:** Conceptualization, Investigation, Methodology. **Chenxu Zhu:** Data curation, Formal analysis, Methodology. **Qifan Yu:** Formal analysis, Methodology. **Li Dong:** Data curation, Formal analysis. **Chen Cui:** Data curation, Methodology. **Zhaofan zhang:** Formal analysis, Methodology. **Jinbo Liu:** Conceptualization, Data curation, Supervision. **Bin Li:** Data curation, Supervision, Writing – review & editing. **Song Chen:** Conceptualization, Project administration, Resources, Supervision, Writing – review & editing.

## Declaration of competing interest

The authors declare that they have no known competing financial interests or personal relationships that could have appeared to influence the work reported in this paper.

## Data Availability

Data will be made available on request.
