# Supplementary material for: Biomimetic double-layered electrospun nanofibrous scaffold with mussel adhesive protein coating and TGF-β3 encapsulation for enhanced tendon-bone healing in rotator cuff tears
Source: Mater Today Bio. 2026 Apr 27;38:103160. doi: 10.1016/j.mtbio.2026.103160 (PMC13145380; doi:10.1016/j.mtbio.2026.103160)
Supplement: Multimedia component 1 [file mmc1.docx]

Supplementary Information

**Biomimetic Double-Layered Electrospun Nanofibrous Scaffold with Mussel Adhesive Protein Coating and TGF-β3 Encapsulation for Enhanced Tendon-Bone Healing in Rotator Cuff Tears**

**Table S1.** Primary antibodies used in this study

| **Antibody** | **Vendor** | **Catalog number** |
| --- | --- | --- |
| **Vinculin** | Abcam, Cambridge, UK | ab129002 |
| **COL Ⅱ (IHC-P)** | Abcam, Cambridge, UK | ab34712 |
| **COL II (WB)** | Abcam, Cambridge, UK | ab188570 |
| **AKT** | Abcam, Cambridge, UK | ab179463 |
| **AKT1 (phosphor S473)** | Abcam, Cambridge, UK | ab81283 |
| **Smad2/3 (WB)** | Abcam, Cambridge, UK | ab202445 |
| **MK-2206 Dihydrochloride** | Selleck, Houston, USA | S1078 |
| **GAPDH** | Abcam, Cambridge, UK | ab181602 |
| **SOX2** | Abcam, Cambridge, UK | ab92494 |

**Table S2.** Primers for RT-qPCR

| **Gene** | **Forward (5’-3’)** | **Reverse (5’-3’)** |
| --- | --- | --- |
| ***Acan*** | CCTGGACAAGTGCTATGCTGG | GCACCACTGACACACCTCGGAA |
| ***Col2a1*** | ACGCTCAAGTCGCTGAACAACC | ATCCAGTAGTCTCCGCTCTTCCAC |
| ***Gapdh*** | GACATGCCGCCTGGAGAAAC | AGCCCAGGATGCCCTTTAGT |


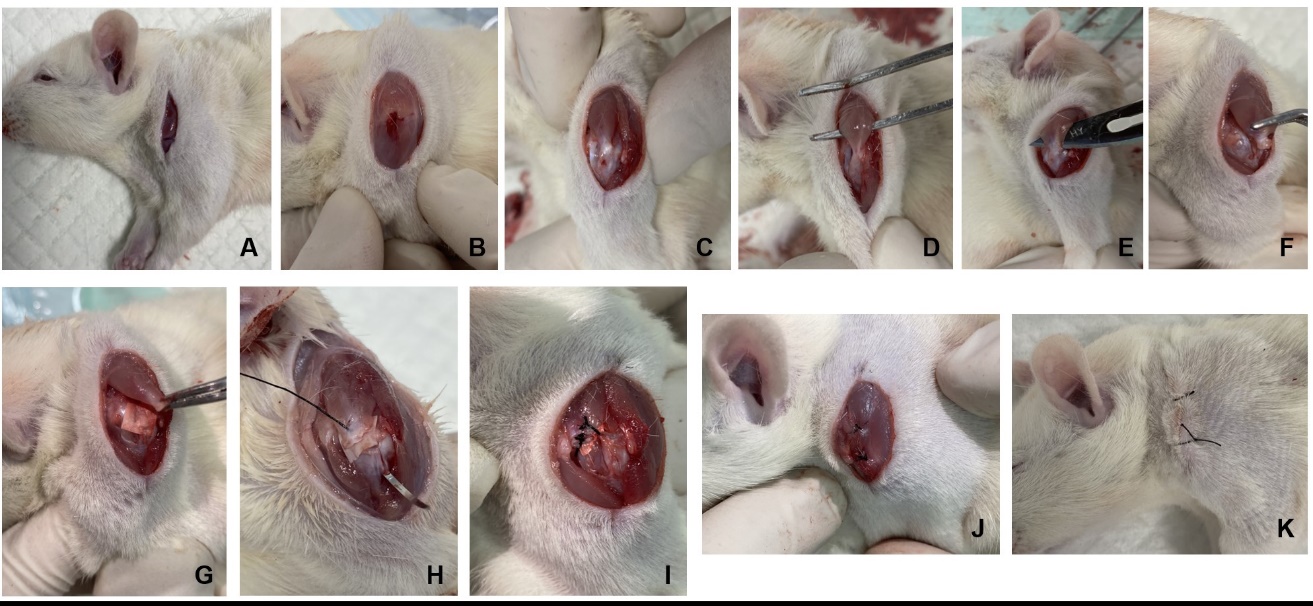


**Fig. S1.** Animal model of rotator cuff tear (RCT) and implantation of scaffolds. (A) The location of the incision: anterolateral to the midline of the shoulder. (B-D) The deltoid muscle and acromioclavicular joint were separated sequentially, and then the rotator cuff tendon was exposed. (E-F) The supraspinatus tendon was dissected at its insertion site on the surface of the humeral great tuberosity to obtain a model of RCT. (G-I) The scaffold was placed between the supraspinatus tendon and the great tuberosity, with the aligned layer facing upward and parallel to the tendon fiber direction. The detached supraspinatus tendon, along with the scaffold, was reattached to the insertion site through a transosseous suturing with 3-0 sutures. (J-K) The deltoid muscle was repaired and the wound was closed.


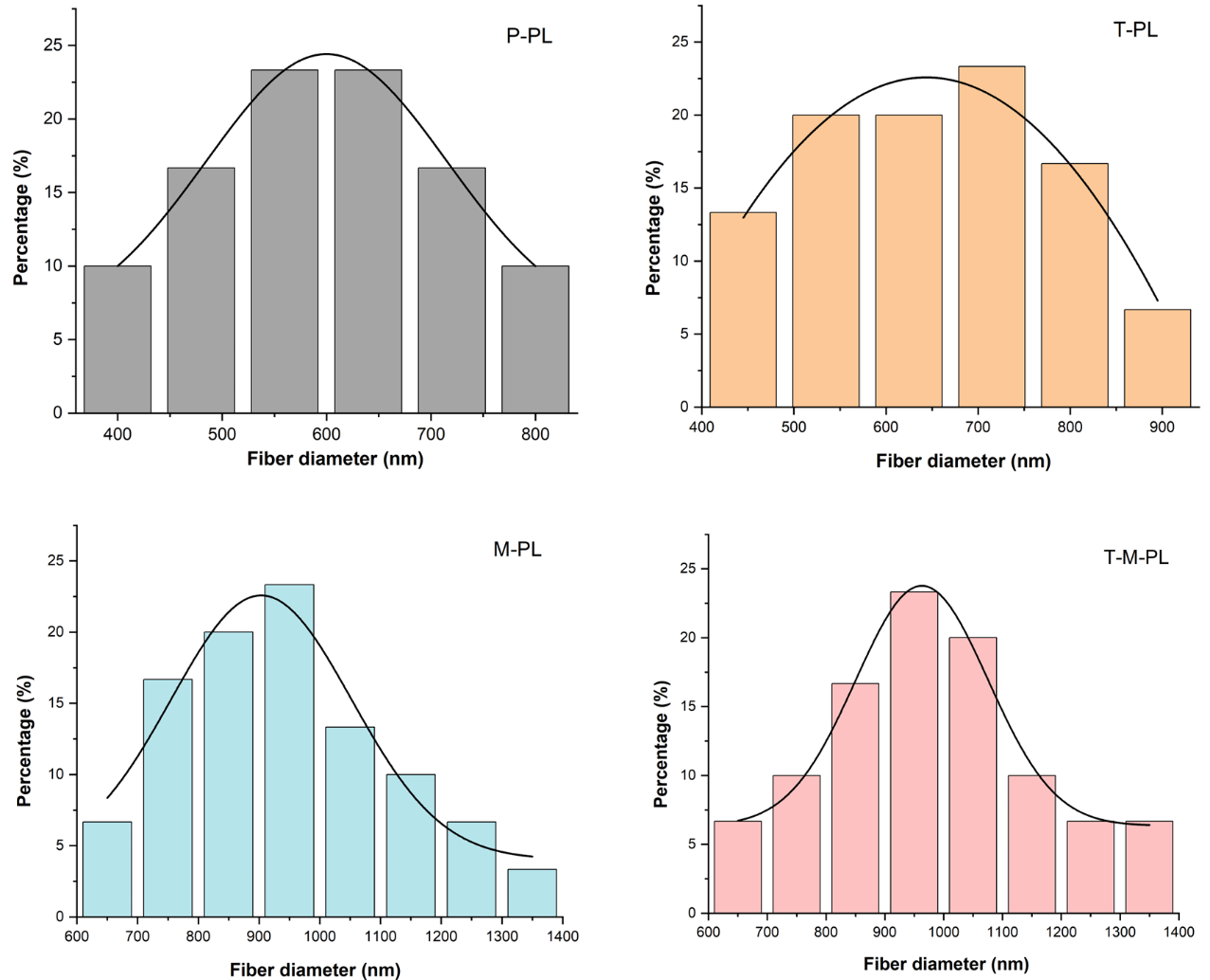


**Fig. S2.** Diameter distribution of different scaffolds


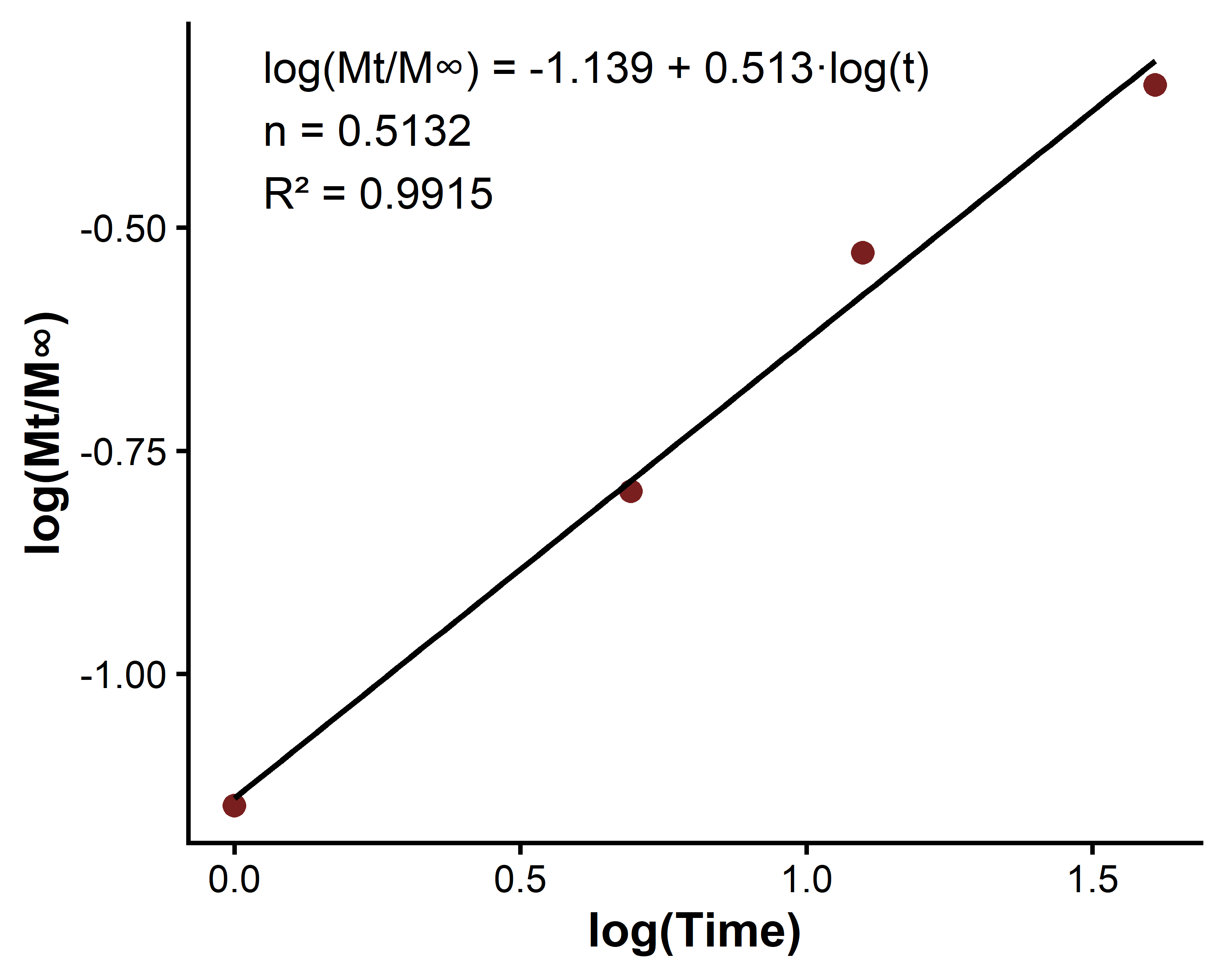


**Fig. S3.** Fitting analysis of TGF-β3 release kinetics follows Korsmeyer-Peppas model


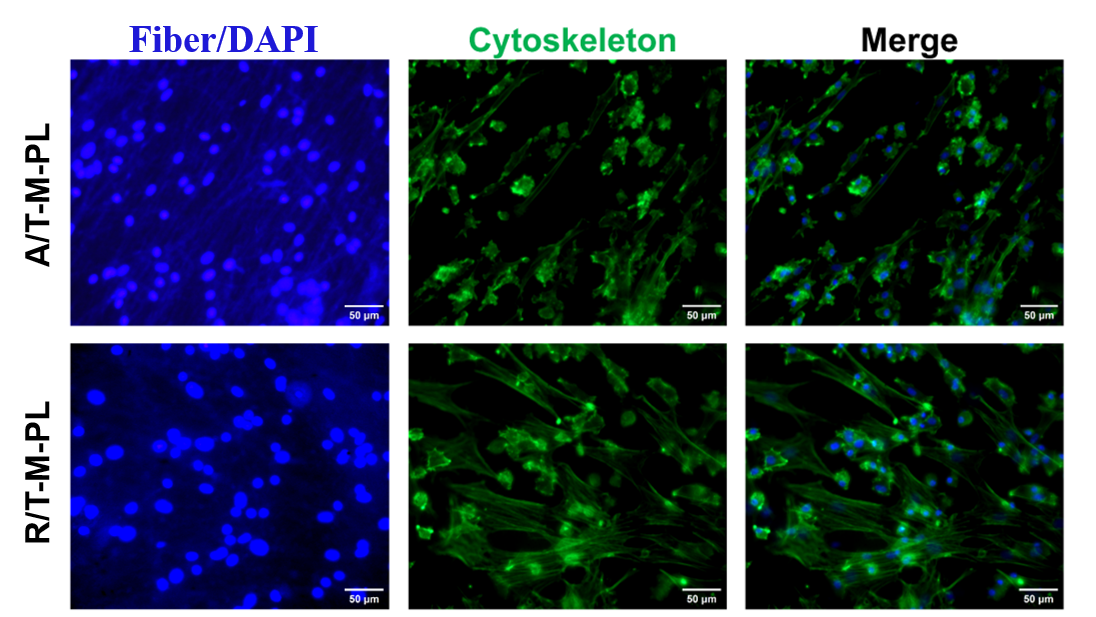


**Fig. S4.** Cytoskeleton staining and fiber orientation.


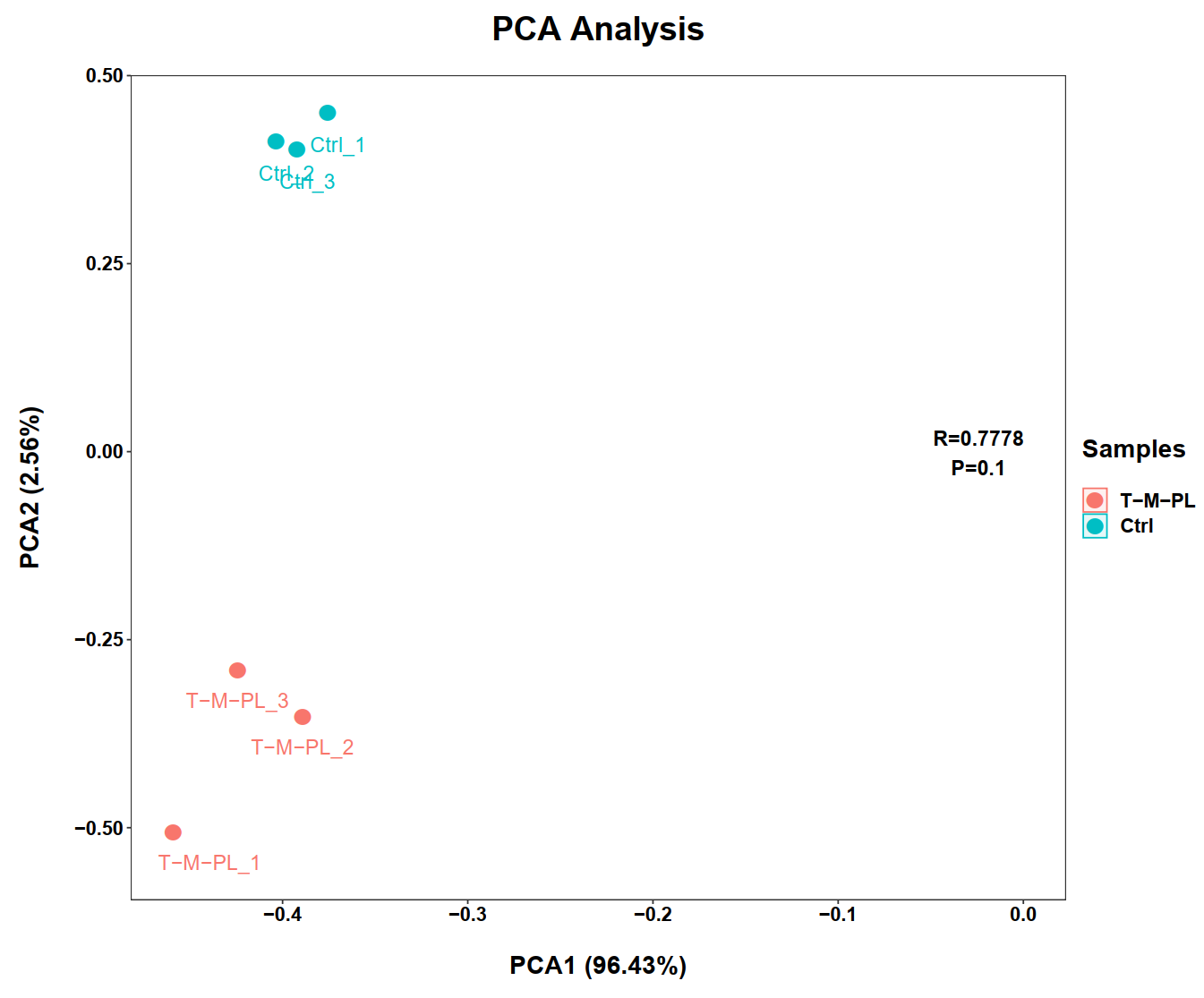


**Fig. S5.** Principal components analysis.


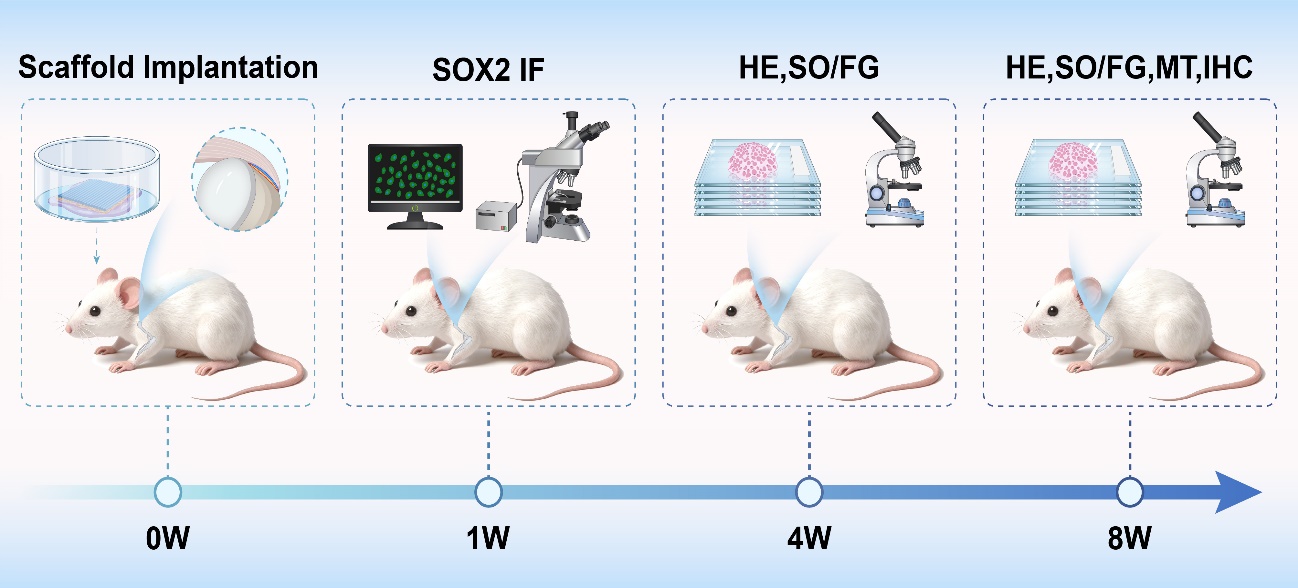


**Fig. S6.** Schematic timeline of the implantation and performance analysis of the scaffold in vivo.
